# Effect of a sensing charge mutation on the deactivation of K_V_7.2 channels

**DOI:** 10.1085/jgp.202213284

**Published:** 2024-01-18

**Authors:** Baharak Mehrdel, Carlos A. Villalba-Galea

**Affiliations:** 1 Department of Physiology and Pharmacology, Thomas J. Long School of Pharmacy, University of the Pacific, Stockton, CA, USA

## Abstract

Potassium-selective, voltage-gated channels of the K_V_7 family are critical regulators of electrical excitability in many cell types. Removing the outermost putative sensing charge (R198) of the human K_V_7.2 shifts its activation voltage dependence toward more negative potentials. This suggests that removing a charge “at the top” of the fourth (S4) segment of the voltage-sensing domain facilitates activation. Here, we hypothesized that restoring that charge would bring back the activation to its normal voltage range. We introduced the mutation R198H in K_V_7.2 with the idea that titrating the introduced histidine with protons would reinstate the sensing charge. As predicted, the mutant’s activation voltage dependence changed as a function of the external pH (pH_EXT_) while modest changes in the activation voltage dependence were observed with the wild-type (WT) channel. On the other hand, the deactivation kinetics of the R198H mutant was remarkably sensitive to pH_EXT_ changes, readily deactivating at pH_EXT_ 6, while becoming slower to deactivate at pH_EXT_ 8. In contrast, the K_V_7.2 WT displayed modest changes in the deactivation kinetics as a function of pH_EXT_. This suggested that the charge of residue 198 was critical for deactivation. However, in a surprising turn, the mutant R198Q—a non-titratable mutation—also displayed a high pH_EXT_ sensitivity activity. We thus concluded that rather than the charge at position 198, the protonation status of the channel’s extracellular face modulates the open channel stabilization and that the charge of residue 198 is required for the voltage sensor to effectively deactivate the channel, overcoming the stabilizing effect of high pH_EXT_.

## Teaser

Mehrdel and Villalba-Galea found that removing the charge of residue R198 in the human K_V_7.2, a critical regulator of electrical excitability, caused a dramatic increase in pH sensitivity on the activity of this channel, with a more robust effect on deactivation.

## Introduction

Voltage-gated, potassium-selective (K_V_) channels from the K_V_7 family are common in the cardiovascular, gastrointestinal, and nervous systems. These proteins constitute the molecular entities responsible for M-currents that were first described as K^+^-currents suppressed by the activation of Muscarinic receptors (Brown and Adams, 1980). Today, we know that K_V_7 channels are regulated in many ways. For instance, K_V_7 activity is tightly regulated by Gq protein-coupled receptors through the manipulation of phosphoinositides concentration, mainly PI(4,5)P_2_ in the plasma membrane (Wang et al., 1998; Jentsch, 2000; Cooper, 2012). Furthermore, K_V_7 channels are also subject to regulation by other lipids, including fatty acids (Taylor and Sanders, 2017; Larsson et al., 2020). Another outstanding property of K_V_7 channels is that their activation is observed at membrane potentials as negative as −60 mV, while a typical K_V_ channel activates at −40 mV or more positive potentials. This feature makes K_V_7 channels critical determinants of the resting membrane potential in diverse cell types, dynamically regulating electrical excitability (Brown and Adams, 1980; Wang et al., 1998; Jentsch, 2000; Cooper, 2012; Linley et al., 2012; Soh et al., 2014; Mastrangelo, 2015; Miceli et al., 2015). Indeed, mutations that impair the normal functioning of K_V_7 channels lead to many types of disorders including benign familial neonatal seizures (Charlier et al., 1998; Singh et al., 1998; Dedek et al., 2001; Wuttke et al., 2008), early onset epileptic encephalopathy (Weckhuysen et al., 2012, 2013; Abidi et al., 2015; Mastrangelo, 2015; Miceli et al., 2015), peripheral nerve hyperexcitability (Dedek et al., 2001; Wuttke et al., 2007), and cardiac arrhythmias and long QT syndromes (Maljevic et al., 2010; Abbott, 2021; Sanguinetti and Seebohm, 2021).

Disruption of M-currents decreases the resting K^+^-conductance in excitable cells of many types, facilitating the triggering of action potentials, thereby boosting excitability (Jentsch, 2000; Maljevic et al., 2010; Cooper, 2012; Abbott, 2021; Sanguinetti and Seebohm, 2021). Accordingly, poorly performing M-currents are seen as the culprit in the generation of out-of-control activity in a cell with K_V_7 channels carrying disruptive mutations. One likely exception to this rule is the case of the K_V_7.2 mutant R198Q. This mutation causes a negative shift in the activation voltage dependence of K_V_7.2 channels (Millichap et al., 2017). Such shift results in an increased activity of the channel at the resting potential when expressed in *Xenopus laevis* oocytes (Millichap et al., 2017). Given the effect of the mutation R198Q on the channel’s voltage dependence, the resting K^+^ conductance should increase in neurons expressing this mutant channel, leading to hypoexcitability. However, R198Q is paradoxically deemed as a mutation linked to infantile spasms with hypsarrhythmia, a form of hyperexcitability-related disorder (Millichap et al., 2017). This led us to hypothesize that the shift of the voltage dependence might not be the reason for this mutation to cause seizures.

It has been reported that in a steady state at the resting potential, the open K_V_7.2 channel becomes more resilient to be closed (Corbin-Leftwich et al., 2016; Villalba-Galea, 2020). Further, it has also been shown that both homomeric K_V_7.2 and heteromeric K_V_7.2/K_V_7.3 channels become even more resilient to closing in the presence of Retigabine at low micromolar concentrations (Corbin-Leftwich et al., 2016). Furthermore, this “resilient-to-close” mode of activity is highly dependent on PI(4,5)P_2_ as the deactivation is facilitated by the partial depletion of this signaling lipid (Villalba-Galea, 2020). These observations suggest that making the channels resilient-to-close (stabilizing the open conformations of the channels) is an essential feature in the physiology of these proteins.

It is well known that altering the sensing charges of a voltage-gated channel can have a strong impact on its voltage dependence and kinetics for activation. In contrast, much less is known about the effect of such alterations on channel deactivation. Thus, we proceeded to investigate whether “gain-of-function” mutations of residue R198 affect channel deactivation. Our study revealed that two non-charged R198 mutations of K_V_7.2 gained another function as their deactivation kinetics became extremely sensitive to extracellular pH (pH_EXT_). We observed that a small reduction of pH_EXT_ (from 7.4 to 7.0) dramatically increased the deactivation rate. This observation was exciting because it has been shown that high neuronal activity can lead to a similar decrease in pH_EXT_ during seizures (Raimondo et al., 2015; Sulis Sato et al., 2017). Considering this notion, we here propose that the mutation R198Q makes the open K_V_7.2 channel less stable when the pH_EXT_ becomes more acidic during periods of high neuronal activity, decreasing the ability of M-currents to curtail electrical excitability, thereby leading to seizures.

## Materials and methods

### Preparation of oocytes and RNA injections

RNA preparation, *Xenopus* oocyte isolation, preparation, and RNA injection were performed using published methods (Villalba-Galea et al., 2008, 2009; Corbin-Leftwich et al., 2016). Animal protocols were approved by the Institutional Animal Care and Use Committees at the University of the Pacific and conform to the requirements in the Guide for the Care and Use of Laboratory Animals from the National Academy of Sciences. Ovarian lobules were surgically harvested from frogs purchased from Xenopus 1. Oocytes were incubated at 16–17°C in a solution of (in mM) 100 NaCl, 1 KCl, 2 CaCl_2_, 1 MgCl_2_ or MgSO_3_, 10 HEPES, 2 pyruvic acid, pH 7.5, and 20–50 mg/l of gentamycin. Results from different batches of oocytes were combined.

### Electrophysiology

Oocytes were injected with 2 ng of each in vitro-transcribed cRNA encoding for the human K_V_7.2 and K_V_7.3 channels. Injected oocytes were incubated at 16–17°C for 2–4 days before recording. The incubation solution was titrated to pH 7.5 with NaOH and contained (in mM) 95 NaCl, 1 NaOH, 2 KCl, 5 HEPES, 1 MgCl_2_, 1.8 CaCl_2_, 1 MgCl_2_, 2 pyruvic acid sodium salt, and 20–50 mg/l of gentamycin.

Potassium currents were recorded using the *Xenopus* oocyte Cut-Open Voltage-Clamp (COVC) technique with a CA-1 amplifier (Dagan Corporation). The external recording solution contained (in mM) 12 KOH, 88 *N*-methyl-*D*-glucamine, 85 methanesulfonic acid, 5 HEPES, 5 MOPS, 5 MES, 0.25 Mg(OH)_2_, and 2 Ca(OH)_2_. The external solution was titrated to pH 6.0, 7.0, 7.4, and 8.0 with methanesulfonic acid or NMDG, accordingly. The intracellular solution contained (in mM) 98 KOH, 2 KHPO_4_, 88 methanesulfonic acid, 10 HEPES, 0.25 Mg(OH)_2_, and 2 EGTA. The intracellular solutions were titrated to pH 7.4 with methanesulfonic acid. Borosilicate glass electrodes (resistance = 0.2–2.0 MΩ) were filled with a solution containing (in mM) 1,000 KCl, 10 HEPES, and 10 EGTA, at pH 7.4 titrated with KOH.

As previously described, voltage control and current acquisition were performed using a USB-6251 multifunction acquisition board (National Instruments) controlled by an in-house program coded in LabVIEW (National Instruments; details available upon request). Current signals were filtered at 100 kHz, oversampled at 500 kHz–2 MHz, and stored at 5–25 kHz for offline analysis. Data were analyzed using custom Java-based software (details available upon request) and Origin 2019/Origin 2023b (OriginLab).

### Exponential fits and weighted average time constant

As described in previous studies (Wickenden et al., 2000; Labro et al., 2012; Villalba-Galea, 2014; Corbin-Leftwich et al., 2016), the following two-exponential function was fitted to the deactivating currents,

IDEACTt=A1e−(t-t0)τ1+A2e−(t-t0)τ2,

where, *A*_1_ and *A*_2_ are the current amplitude associated with each component, and τ_1_ and τ_2_ are the corresponding time constants. The parameter *t*_0_ is the time at which deactivation starts. Fittings were done using Origin 2019 (OriginLab). When needed, the deactivation weighted average time constant (τ_DEACT_) was calculated as

$$\tau_{DEACT}=\frac{A_{1}\tau_{1}+A_{2}\tau_{2}}{A_{1}+A_{2}}\text{.}$$

*T* tests were calculated for statistical analysis of the time constants.

It is important to highlight that the two-exponential equation was not derived from a comprehensive kinetic model describing the activity of K_V_7 channels. Instead, it was selected because it can tightly trace deactivating currents. Consequently, the parameters yielded from fitting the equation to such currents can only provide a temporal description of the deactivation process. Meaningful assignment of each individual parameter to any physical process underlying the activity of the channel under study is therefore extremely limited and even inadequate.

### Fermi–Dirac distribution, weighted V_1/2_, and total apparent charge

The weighted V_1/2_ was calculated from the values yielded by the fit of a double Fermi–Dirac distribution to the I_TAIL_–V_ACT_ plots. The double Fermi–Dirac distribution is defined as

ITAIL=A11+e−z1F(VACT−V1)RT+A21+e−z2F(VACT−V2)RT,

where *A*_1_ and *A*_2_ represent the amplitude of each of the components, *z*_1_ and *z*_2_ the apparent sensing charge associated with each component, and *V*_1_ and *V*_2_ are the half-maximum potential for each of the components. *F*, *R*, and *T* are the Faraday constant, the universal gas constant, and the absolute temperature, respesectively. From here, the weighted V_1/2_ is calculated as

$$weighted\;V_{1/2}=\frac{A_{1}V_{1}+A_{2}V_{2}}{A_{1}+A_{2}}\text{.}$$

The total apparent charge is given by

$$z_{apparent}=z_{1}+z_{2}\text{.}$$

### Activation kinetic

To quantify the activation kinetics, we used an empirical equation that assumes that *n* number of independent subunits are required to activate the conductance of the channels and that each subunit is activated in two steps. This is similar to what has been previously proposed for *Shaker* (Zagotta et al., 1994). We assumed the existence of a two-step process given that the activation displayed a fast and a slow component (e.g., Fig. 3 A). A two-step activation is represented by the sum of two exponential functions, and the requirement of >1 independent subunit for activation is represented by the power to *n*. The resulting equation is defined as

$$I_{ACT}=A{\left[f_{1}\left(1-e^{-\frac{t}{\tau_{FAST}}}\right)+\left(1-f_{1}\right)\left(1-e^{-\frac{t}{\tau_{SLOW}}}\right)\right]}^{n}\text{,}$$

where A is the maximum amplitude for the currents, f1 represents the fractional contribution of the fast component, *t* is time, and *τ*_*FAST*_ and *τ*_*SLOW*_ are the time constants of the fast and slow component, respectively.

### Molecular biology

The constructs human KCNQ2 and KCNQ3 in the expression vector pTLN, encoding K_V_7.2 and K_V_7.3 channels, were linearized with MluI and HpaI (New England Biolabs), respectively. The linearized K_V_7-encoding cDNA was transcribed using an SP6 RNA polymerase kit (Ambion mMessage mMachine; Thermo Fisher Scientific). Mutations were introduced using the Q5 Site-Directed Mutagenesis Kit (New England Biolabs).

### Structural model

A cryo-EM-derived structural model of K_V_7.2 bound to calmodulin (PDB ID 7CR3; Li et al., 2021) was incorporated in a POPC bilayer containing PI(4,5)P_2_ using the online tool Charmm-Gui. Then, the model was minimized and equilibrated to 298.15^o^ K using NAMD. A 10-ns simulation at 298.15^o^ K was performed in the absence of any electrical field. No restrictions were imposed on the structure during the simulation. The simulation was performed through an allocation grant from ACCESS.

### Statistical analysis

*T* tests were performed to determine significant differences between values by using the mean, SD, and number of observations to calculate P values. The confidence was set at 95%. *T* tests were performed using pH_EXT_ 7.4 as a reference in figures plotting a parameter as a function of another parameter at different pH_EXT_ (e.g., Fig. 3, D–I). *T* tests were performed using Origin 2023b (OriginLab).

### Online supplemental material

**Figure S1.**
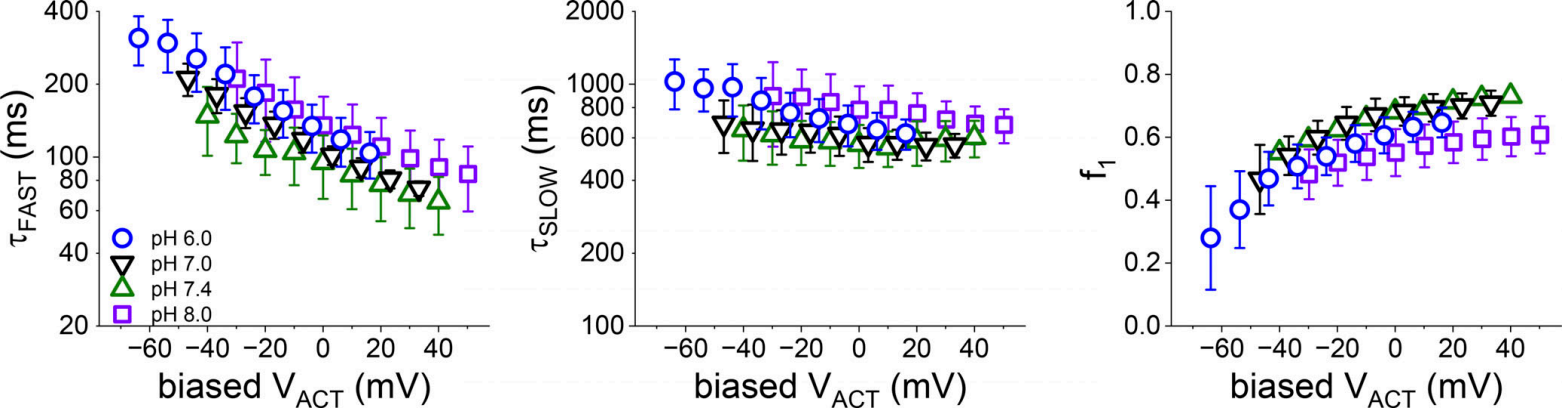
**The mutant R198H displayed a shift in voltage dependence of activation that was about −17 mV per unit of pH**_**EXT**_**.** If the change in voltage dependence is due to a purely electrostatic effect on the VSD, then the effect on the kinetic of activation is due to a local electrostatic bias. Accordingly, the plots in Fig. 3, G–I, were replotted changing the values of the X-axis at a rate of −17 m V/pH_EXT_, using the V_ACT_ values for the plot at pH_EXT_ 7.4 as a reference, In doing so, we observed that the plots in Fig. 3, G–I, tend to overlap, strongly suggesting that the difference in the parameters t_FAST_, t_SLOW_, and f_1_ are due to an electrostatic effect from the protonation.

To account for potential electrostatic bias on the kinetic of activation, Fig. S1 shows the plots in Fig. 3, G–I, were replotted by changing the values of the X-axis at a rate of −17 mV/pH_EXT_, using the V_ACT_ values for the plot at pH_EXT_ 7.4 as reference.

## Results

### Protonation of the R198H mutant biases voltage sensitivity for current activation

**Figure 1.**
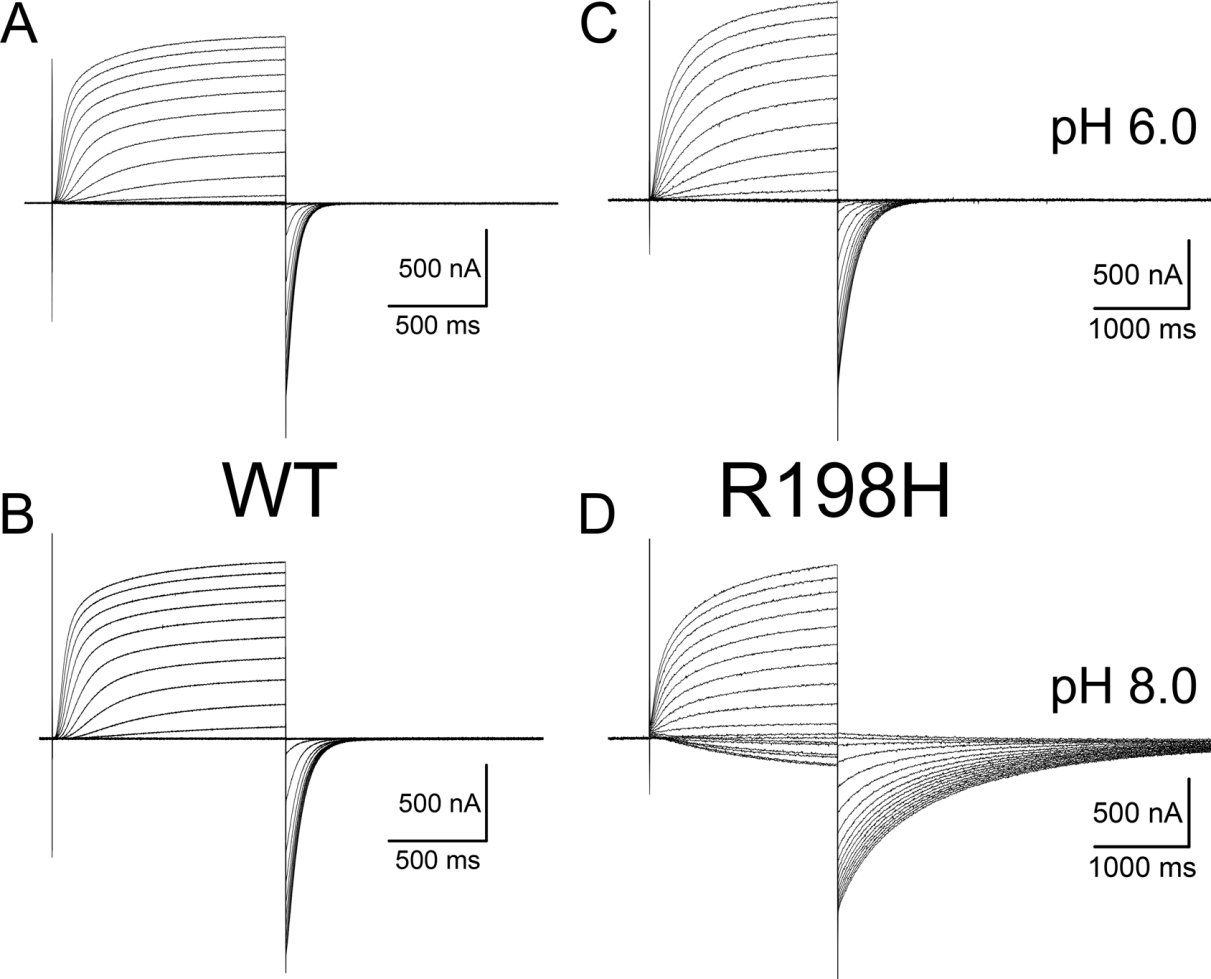
**Currents recording from oocytes expressing K**_**V**_**7.2 channels WT and mutant R198H. (A and B)** WT currents were activated with pulses ranging between −120 and +60 mV from a H.P. of −90 mV. **(C and D)** Equivalent recordings from oocytes expressing the R198H mutant with pulses ranging between −130 and +40 mV. A 5-s prepulse to −120 mV to these recordings as the voltage dependence of the mutant channel was shifted to more negative potentials with respect to WT. To evaluate the effect of external pH_EXT_, we recorded currents at external pH (pH_EXT_) ranging between 6.0 (A and C) and 8.0 (B and D). Remarkably, the rate of deactivation of R198H unambiguously decreased at pH 8.0 with respect to pH 6.0, while WT showed more modest changes.

Mutating the arginine 198 to a glutamine (R198Q) causes a negative shift in the activation voltage dependence of K_V_7.2 channels (Millichap et al., 2017; Edmond et al., 2022). To assess the effect of positive charges on residue 198 of the human K_V_7.2, we proceeded to study the effect of the mutation R198H. Similar to what was observed with the residue R217 in Ci-VSP (Villalba-Galea et al., 2013), we were expecting to change the activation voltage dependence of the channel as a function of the protonation status of the introduced histidine if this manipulation effectively changed the charge of the residue. Following this approach, we recorded K^+^ currents from oocytes expressing either wild-type (WT) human K_V_7.2 and changed the extracellular pH (pH_EXT_) between 6.0 and 8.0. Using the COVC technique, we applied voltage pulses ranging from −120 to +60 mV from a holding potential (H.P.) of −90 mV; deactivation was driven at −105 mV. In these recordings, we noticed that the activation of the K_V_7.2 WT was almost impervious to changes in pH_EXT_, showing small variations in kinetics (Fig. 1, A and B). However, the deactivation process was slightly more sensitive to pH_EXT_ changes, slowing down at alkaline pH_EXT_ (Fig. 1 B) with respect to acidic pH_EXT_ (Fig. 1 A). For the mutant R198H, we applied a similar recording protocol. However, a 7-s prepulse to −120 mV preceded the test pulses, driving the closure of this mutant which displayed unambiguous activity at −90 mV at pH_EXT_ above 7.0. When voltage steps above −90 mV were applied, the activation kinetics seemed almost unaltered by changes in pH_EXT_. Surprisingly, however, the deactivation kinetics was remarkably dependent on the pH_EXT_. Indeed, increasing pH_EXT_ to 8.0 made the closing of the K^+^ current (Fig. 1 D) unambiguously slower than that at pH_EXT_ 6.0 (Fig. 1 C).

**Figure 2.**
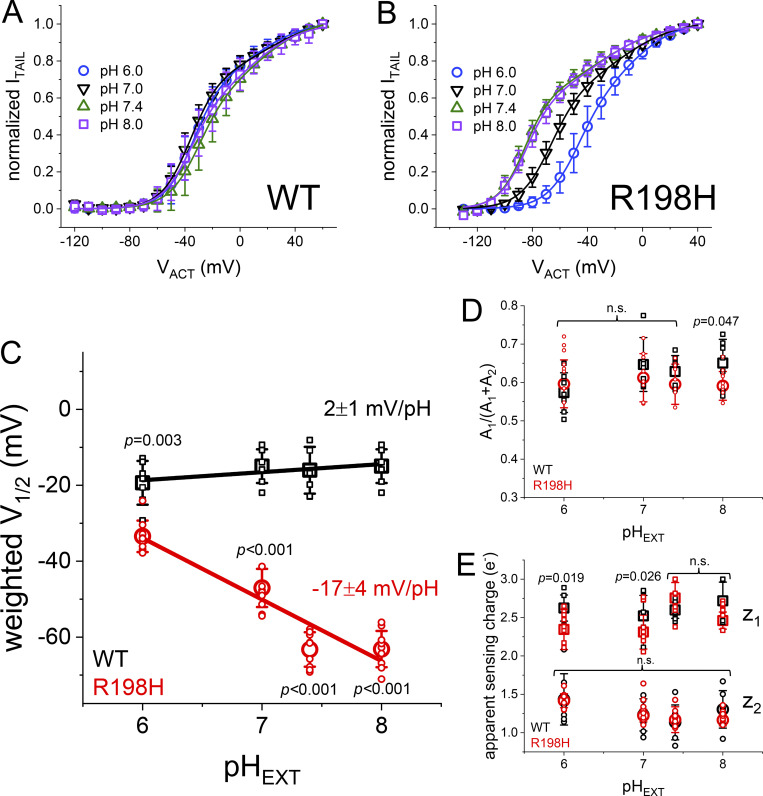
**Average normalized amplitude of deactivating K**^**+**^**-currents (“tail” currents, I**_**TAIL**_**) plotted against the activating membrane potential (V**_**ACT**_**). (A)** From oocytes expressing WT K_V_7.2, the amplitude of I_TAIL_ was normalized by its maximum peak current value in each individual recording. Then, the normalized I_TAIL_ amplitudes were averaged and plotted as a function of V_ACT_. The plots from recordings at pH_EXT_ ranged from 6.0 to 8.0. **(B)** Equivalent I_TAIL_–V_ACT_ plots were generated from the recording of K^+^-current in oocytes expressing the mutant R198H. The I_TAIL_–V_ACT_ plot shifted about −40 mV at pH_EXT_ 8.0 compared with pH_EXT_ 6.0. **(C)** A double Fermi–Dirac equation was fitted to I_TAIL_–V_ACT_ plots from each experiment (see Materials and methods) and the weighted half-maximum of the I_TAIL_–V_ACT_ curves (V_1/2_) and plotted as a function of pH_EXT_. To quantify the pH_EXT_-dependence of V_1/2_, the V_1/2_–pH_EXT_ plots were fitted with a linear regression, yielding values of 2 ± 1 (mean ± SE, *n* = 7) and −17 ± 4 (*n* = 7), for WT and R198H, respectively. **(D and E)** Other two parameters derived from the Fermi–Dirac distribution were plotted against pH_EXT_, namely the relative weight of the most negative component of the distribution (D) and the apparent charges associated with each component (E).

First, to further understand the effect of pH_EXT_ on the activation of the mutant R198H, we plotted the normalized maximum amplitude of deactivating currents (I_TAIL_) as a function of the amplitude of the activating membrane potential (V_ACT_). The I_TAIL_–V_ACT_ plots showed a shift of about −40 mV at pH_EXT_ 8.0 (Fig. 2 B, purple squares) with respect to pH_EXT_ 6.0 (Fig. 2 B, blue circle). In contrast, such changes in pH_EXT_ had a modest effect on the WT channel activation (Fig. 2 A). This strongly suggested that the protonation of the histidine in position 198 was effectively altering voltage sensing during the activation of this channel. To quantify the effect of pH_EXT_ on voltage dependence, we fitted a double Fermi–Dirac function to the I_TAIL_–V_ACT_ plots, not making any assumption of the underlying mechanism for voltage sensing. The Fermi–Dirac function is what is typically referred to as the “Boltzmann distribution.” This latter name is a misnomer (Villalba-Galea and Chiem, 2020). Nonetheless, we calculated the average weighted half-maximum potential (V_1/2_) for each pH_EXT_ from the parameters yielded by the fit. We observed that the weighted V_1/2_ values for the WT channels were modestly altered by changes in pH_EXT_ (Fig. 2 C, black squares), displaying a 2 ± 1 mV change per unit of pH_EXT_ (Fig. 2 C, black line: linear regression). In contrast, the weighted V_1/2_ for the R198H mutant shifted to more negative potentials (Fig. 2 C, red circles), displaying a −17 ± 4 mV change per unit of pH_EXT_ (Fig. 2 C, red line: linear regression). From the fits, we also observed that the apparent sensing charges (Fig. 2 D) and the fractional contributions of each component (Fig. 2 E) were barely altered as a function of pH_EXT_. Although these values for apparent charge cannot be used to calculate the actual sensing charge involved in voltage sensing (Bezanilla and Villalba-Galea, 2013), the fact that they are not altered strongly suggests that the intrinsic ability of the VSD to sense the electrical field was not modified by changes in pH_EXT_. In other words, changes in the protonation status of the introduced histidine seemed to only produce an electrostatic effect, biasing the electrical field at the resting/deactivated state of the VSD.

### Effect of protonation on the activation kinetics

**Figure 3.**
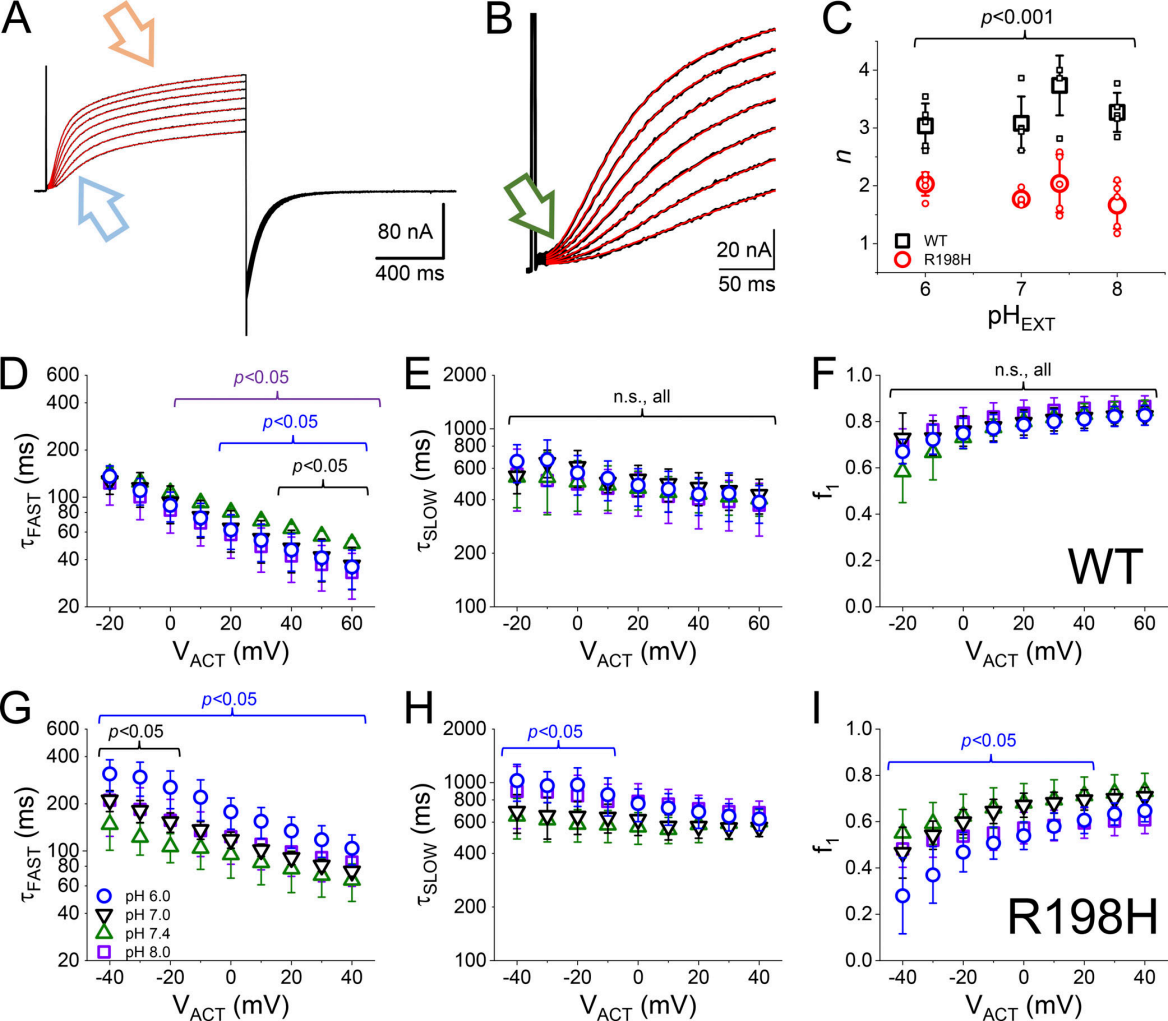
**Kinetics of K**^**+**^**-current activation. (A)** The activating current displayed two kinetic components, hereafter “fast” (teal arrow) and “slow” (orange arrow). **(B)** Also, the activation displayed a lag phase (green arrow). To accommodate these features, an *n*-powered double exponential function (red traces) was fitted to these activating K^+^-currents (black traces). **(C)** The index *n* was different between WT and the mutant R198H, yet, it also seemed impervious to changes in pH_EXT_. **(D and G)** Average time constant of the fast component (*τ*_FAST_) as a function of the activating potential (V_ACT_) for both WT and R198H (*n* = 5, each). **(E and H)**. Average time constant as a function of the activating potential (V_ACT_) for the slow component (*τ*_SLOW_) for both WT and R198H (*n* = 5, each). **(F and I)** Fraction of the fast component for the activation process for both WT and R198H (*n* = 5, each). Statistical test performed using pH_EXT_ 7.4 as reference. *T* test confidences are color coded.

To further understand the effect of the mutation R198H on the activation of K_V_7.2 channels, we proceeded to fit an *n*-powered two-exponential function to the activating currents as a function of time (Fig. 3 A). The two exponentials were used to fit the fast and slow components in the activation of these K^+^ currents (Fig. 3 A, teal and orange arrows, respectively). Furthermore, the function was elevated to the *n*-th power to be able to fit the lag phase of activation (Fig. 3 B, green arrow). It is generally expected that the parameter *n* would tend to be close to 4 given the tetrameric nature of the K_V_ channels (Zagotta et al., 1994)—this is indeed an expectation set since the 1950s, starting with the work of Hodgkin and Huxley in the squid axon (Hodgkin and Huxley, 1952). Yet, we observed that the values for *n* were around 3 for K_V_7.2 WT and 2 for the mutant R198H. These observations suggested that the activation of less than four subunits is required for the ionic conduction to be elicited in these channels. This latter interpretation is consistent with the elegant work from the laboratory of Dr. Rene Barro-Soria (University of Miami), which suggests that not all VSD are to be activated to open the K_V_7.2 channels conduction (Barro-Soria, R., personal communication). This is an intriguing idea that we did not further explore as it was beyond the scope of this work.

Focusing back on the kinetic of activation, the time constants for the fast and slow components for the WT channel, τ_FAST_, and τ_SLOW_, respectively, were almost impervious to changes in pH_EXT_ (Fig. 3, D and E). Furthermore, the contribution of the fast component to the activation (*f*_1_) was also resilient to changes in pH_EXT_ (Fig. 3 F). This indicated that the activation process of the WT channel had a low pH_EXT_ sensitivity. In contrast, the activation of the mutant R198H was unambiguously modulated by pH_EXT_. The fast component seemed slower at pH_EXT_ 6 (Fig. 3 G, blue circles), while the slow component seemed less sensitive to changes in pH_EXT_ (Fig. 3 H). Furthermore, the fractional contribution of the fast component seemed to decrease at pH_EXT_ 6.0 compared with more alkaline conditions (Fig. 3 I). Although the rates changed with pH_EXT_, it is important to consider that the voltage dependence of activation also changes with pH_EXT_ at an apparent rate of about −17 mV per unit of pH_EXT_. This would make the voltage dependence of the activation rates at pH_EXT_ 6.0 offset by 34 mV with respect to pH_EXT_ 8.0. This indicated that the difference observed in the time constants was likely due to the change in voltage dependence. Indeed, shifting the time constants versus potential curves for the mutant R198H (Fig. 3, G–I) made the difference between these curves to be more modest (Fig. S1). This led us to conclude that changes in electrostatic bias were the main effect of protonation on the kinetics of activation.

### Effect of pH_EXT_ on deactivation

**Figure 4.**
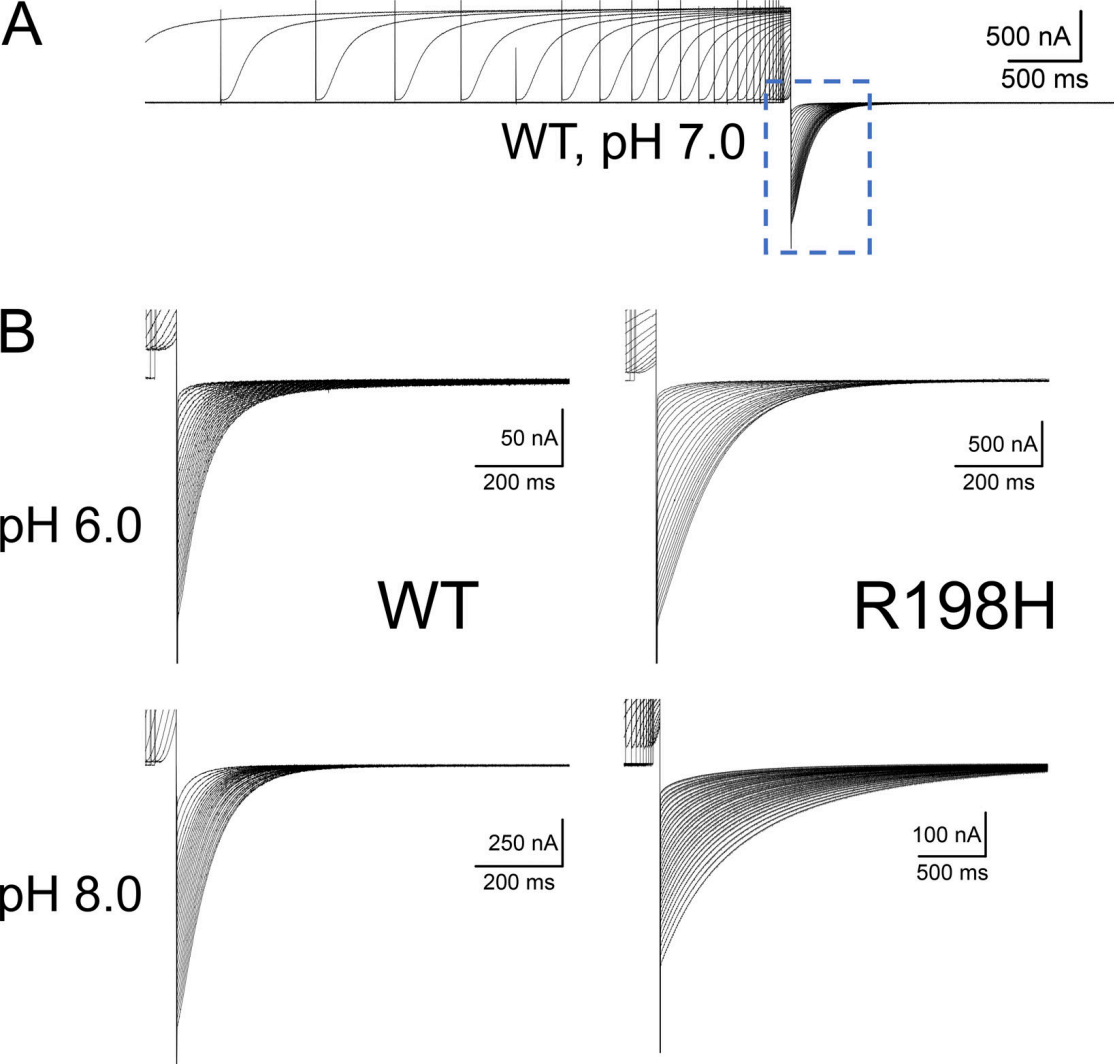
**Deactivating of the K**^**+**^**-current from *Xenopus* oocytes expressing K**_**V**_**7.2 channels WT and R198H at pHEXT 6.0 and 8.0. (A)** Example of K^+^-currents activated with a +40-mV pulse of exponentially increasing duration followed by deactivation at −105 mV. Deactivating phase inside dashed square. **(B)** Detailed deactivating K^+^-currents in oocytes expressing WT (left) and the mutant R198H (right) at pH_EXT_ 6.0 (top) and 8.0 (bottom). Notice that the difference in the time scale for deactivation of the mutant R198H is at pH_EXT_ 8.0.

To evaluate the effect of pH_EXT_ on deactivation, we proceeded to study the deactivation kinetics as a function of activation using a previously established paradigm (Corbin-Leftwich et al., 2016; Villalba-Galea, 2020). Briefly, from a holding potential of −90 mV, we evoked K^+^ currents by applying +40-mV pulses of variable durations (Fig. 4 A), followed by a deactivating pulse to −105 mV (Fig. 4 A, dashed square). The deactivation kinetics was assessed during this second pulse to negative potentials. For the mutant R198H, this protocol was changed given that the currents were already activated at −90 mV. So, we set the H.P. to be close to the nominal reversal potential for K^+^ −50 mV (we had 12 mM K^+^ in the external solution and 100 mM K^+^ in the internal medium). The reason for setting the H.P. to this voltage was to avoid the accumulation of K^+^ in the extracellular side of the membrane. From this H.P., a 1.5-s ramp to −120 mV followed by a 7-s pulse to −120 mV was applied to close the channel. After that, a +40-mV pulse was applied to activate the channels. Then, deactivation was driven at −105 mV.

**Figure 5.**
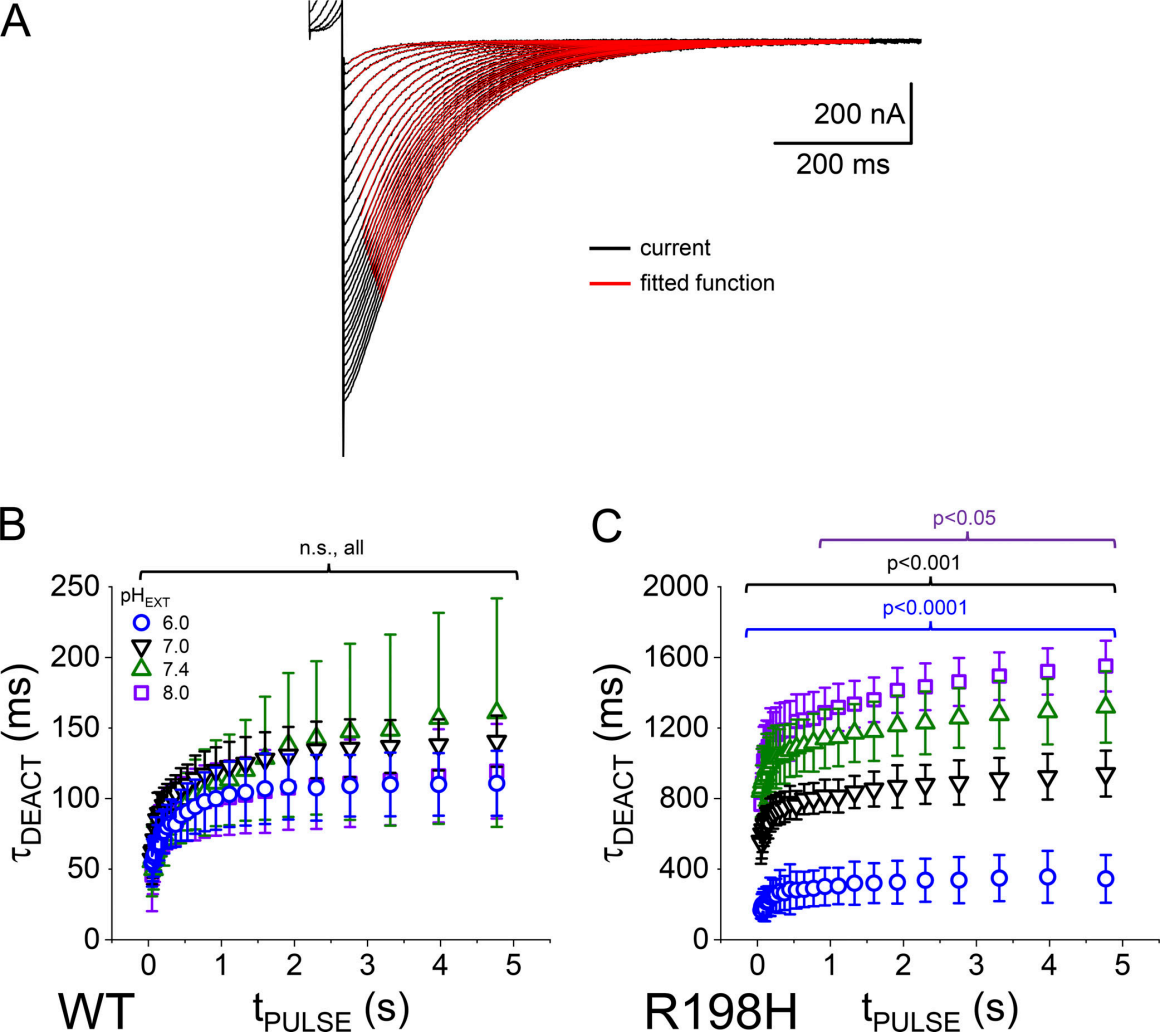
**Analysis of the deactivation time constant. (A)** A two-exponential function was fitted to the deactivating K^+^-currents. The monotonically decaying phase of the deactivating currents was fitted with a double exponential function (red traces); there is a small yet unambiguous lag phase at the beginning of the deactivation currents. **(B and C)** The average deactivation time constants (*τ*_*DEACT*_) were plotted as function of the duration of the activating pulse (t_PULSE_) for oocytes expressing WT K_V_7.2 (B, *n* = 9) and the mutant R198 (C, *n* = 16 for pH_EXT_ 6.0, *n* = 11 for pH_EXT_ 7.0, *n* = 8 for pH_EXT_ 7.4, and *n* = 6 for pH_EXT_ 8.0). Statistical test was performed using the pH_EXT_ 7.4 as reference. *T* test confidences are color-coded.

As reported before, the deactivation kinetics of K_V_7.2 channels become slower as a function of the activating pulse durations (t_PULSE_; Villalba-Galea, 2020). In the case of WT, the slowdown of the deactivation kinetics was slightly affected by changing pH_EXT_ (Fig. 4 B, left panels). In contrast, deactivation of the mutant R198H was remarkably slower at alkaline pH_EXT_ with respect to acidic conditions and with respect to WT (Fig. 4 B, right)—notice time scale. To quantify the deactivation kinetics, a double exponential function was fitted to the monotonically decaying phase of the deactivating currents as previously described (Corbin-Leftwich et al., 2016; Villalba-Galea, 2020; Fig. 5 A, red traces). The weighted average time constants (τ_DEACT_) were then plotted against t_PULSE_ (Fig. 5). The τ_DEACT_–t_PULSE_ plot shows that deactivation became slower as the activating pulse was longer. As been previously reported (Corbin-Leftwich et al., 2016; Villalba-Galea, 2020), we observed that the slowdown of deactivation happens in two phases: an initial phase in which the deactivation time constant more than doubles t_PULSE_ is <1 s and a late phase in which the deactivation changes in a more modest manner as a function of t_PULSE_. For WT, we observed great variability in the second phase. Yet, changing pH_EXT_ did not produce an unambiguous alteration of the τ_DEACT_–t_PULSE_ plot (Fig. 5 B). In contrast, the τ_DEACT_–t_PULSE_ relationship for the mutant R198H was extremely sensitive to pH_EXT_ (Fig. 5 C). In this latter case, increasing pH_EXT_ caused a further decrease in τ_DEACT_, reaching a sevenfold increase in the deactivation time constant at pH_EXT_ 8.0 with respect to pH_EXT_ 6.0. Like in previous reports, this suggested that the slowdown of the deactivation was due to a transition into a more stable conductive/activated conformation of the channel and that such conformation was further stabilized in the R198H mutant at a higher pH_EXT_.

### Absence of a positive charge in position 198 enhances K_V_7.2 channel’s pH_EXT_ sensitivity

**Figure 6.**
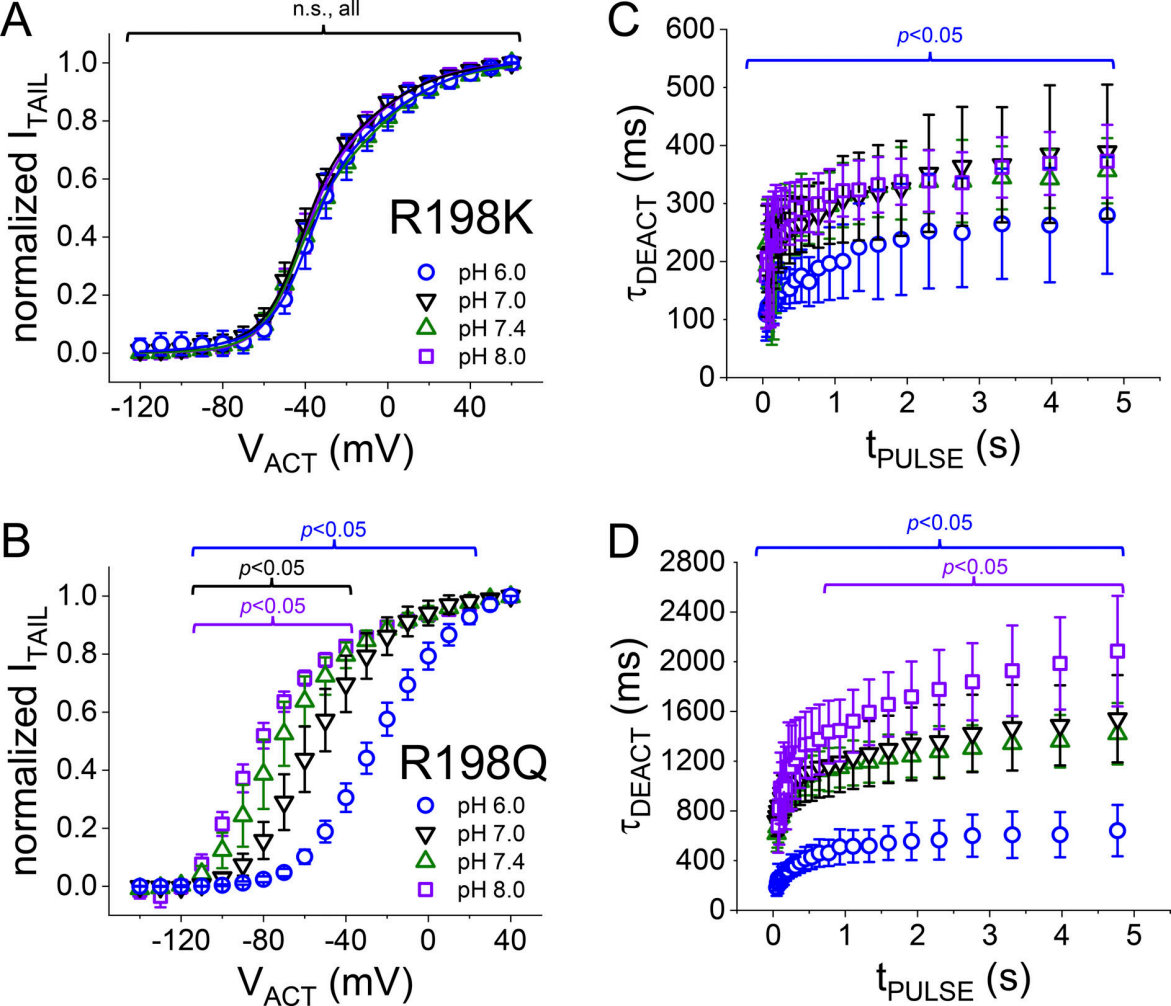
**Activation voltage dependence and deactivation kinetics of the mutants R198K and R198Q. (A and B)** I_TAIL_–V_ACT_ plots generated from K^+^-currents recorded from oocytes expressing the mutants R198K (A) and R198Q (B) at pH_EXT_ ranging from 6.0 to 8.0. **(C and D)**. Deactivation average time constant (τ_DEACT_) for the mutants R198K (*n* = 8) (C) and R198Q (D) also at pH_EXT_ 6.0 through 8.0. Statistical test performed using the WT as reference. *T* test confidence intervals are color-coded; *n* = 8 for pH_EXT_ 6.0, *n* = 11 for pH_EXT_ 7.0, *n* = 12 for pH_EXT_ 7.4, and *n* = 7 for pH_EXT_ 8.0.

To assess the need for a charge at position 198 to prevent enhancing K_V_7.2 channels’ pH_EXT_ sensitivity, we engineered two additional mutations. The first one, R198K, introduced a residue that would be always charged within the range of pH_EXT_ considered in this study. We observed that the weighted V_1/2_ for the mutant was −26 ± 3 mV (*n* = 7), which was negatively shifted with respect to WT with a weighted V_1/2_ of −18 ± 5 mV (*n* = 7). Although there was a shift in the voltage dependence of this mutant channel at pH_EXT_ 7.4 (P = 0.0034), such property was almost impervious to changes in pH_EXT_ like the WT (Fig. 6 A). This strongly suggested that the charge borne by the lysine at position 198 was able to prevent changes in the channel’s activation by pH_EXT_.

Coming full circle, the second mutation considered was R198Q. This is a clinically found mutation linked to spasms and seizures (Millichap et al., 2017). The mutation R198Q shifts the voltage dependence for activation of K_V_7.2 channels toward negative potentials. As expected, we observed the weighted V_1/2_ to be at −64 ± 7 mV and −64 ± 3 mV (*n* = 7 for both) at pH_EXT_ of 7.4 and 8.0, respectively. Yet, to our surprise, the weighted V_1/2_ positively shifted to −52 ± 7 mV and −21 ± 6 mV (*n* = 7 for both) at pH_EXT_ 7.0 and 6.0, respectively (Fig. 6 B). This strongly suggested that, in the absence of a charged residue, the channel voltage-dependence becomes pH_EXT_ sensitive independently of the protonation status of residue 198.

To further evaluate the effect of the mutations R198K and R198Q, we studied the deactivation kinetics of the K^+^-currents. Like WT, the mutant R198K displayed fast deactivating currents at all pH_EXT_ evaluated. We observed slight changes in the kinetic of deactivation, with the slowest kinetics being at pH_EXT_ 8.0 (*n* = 7; Fig. 6 C). In contrast, the mutant R198Q displayed a fivefold difference in the deactivation kinetics between pH_EXT_ 6.0 and 8.0 (Fig. 6 D). These observations strongly suggested that a charged residue in position 198 allows the channels to overcome pH_EXT_-related effects on the deactivation kinetics. We are yet to understand what residues participate in this enhancement in pH_EXT_-sensitivity. Nonetheless, our results seem to indicate that having a positively charged residue at position 198 allows the channels to readily close.

### R198 mutants remain pH-sensitive when coexpressed with K_V_7.3

The effect of the mutation R198Q on the activation voltage dependence of K_V_7.2 is mitigated by the coexpression of K_V_7.3 (Millichap et al., 2017). Thus, we proceeded to evaluate how the presence of K_V_7.3 affected the acquired pH_EXT_ sensitivity. To do so, we performed the same type of recordings described above from oocytes coexpressing the K_V_7.3 channel subunit.

**Figure 7.**
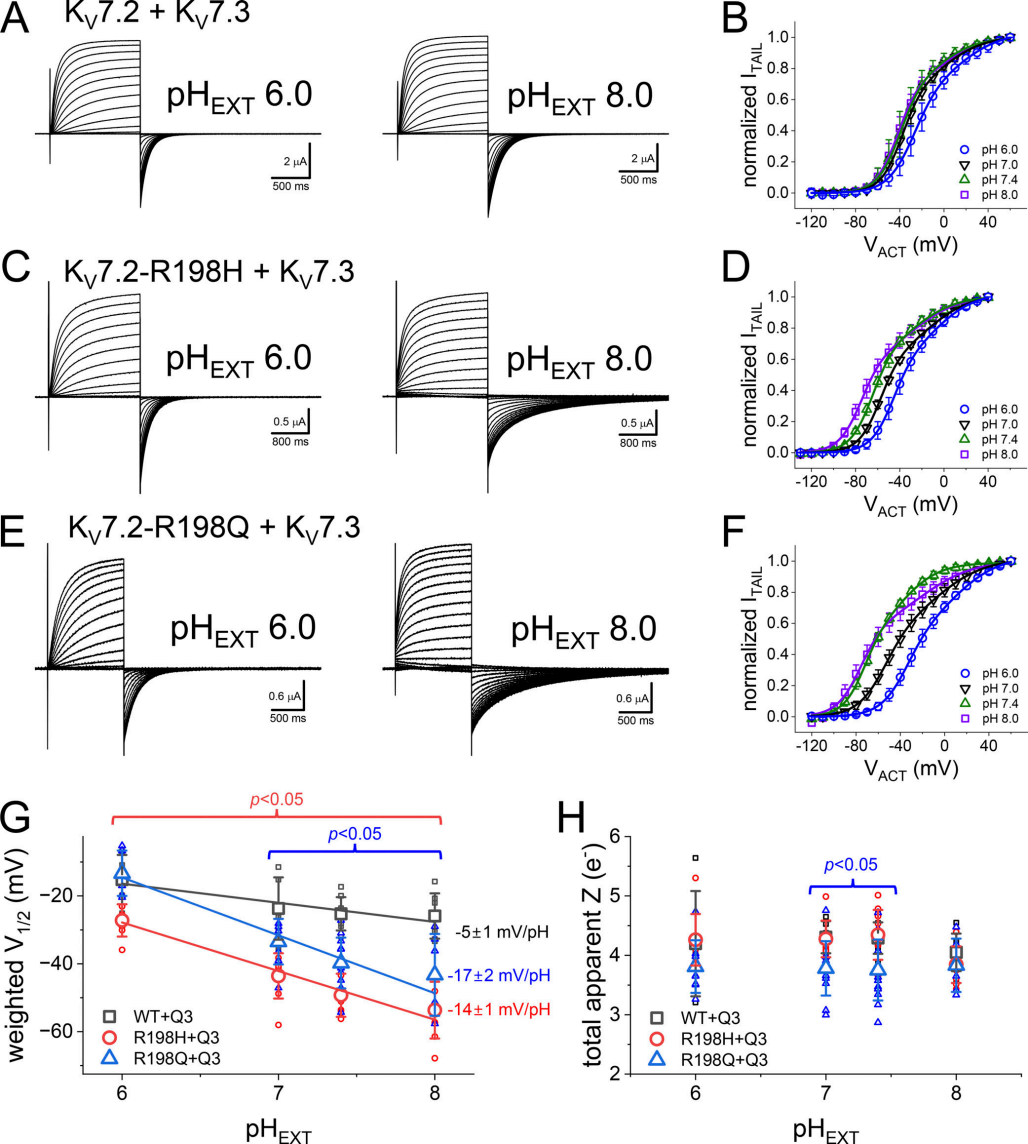
**Effect of the coexpression of K**_**V**_**7.3 on the deactivation kinetics at different pH**_**EXT**_**. (A)** K^+^ current recordings from oocytes expressing the WT K_V_7.2 channel coexpressed with K_V_7.3. The recordings were performed at pH_EXT_ 6.0 (left) and 8.0 (right). The holding potential was set to −90 mV, and the amplitude of the activating test pulses (V_ACT_) ranged from −120 to +60 mV. Deactivation was driven at −105 mV. **(B)** Average I_ACT_–V_ACT_ plots at pH_EXT_ ranging from 6.0 to 8.0. **(C)** K^+^-currents from oocytes coexpressing the mutant R198H with K_V_7.3. In this case, the holding potential was set at −50 mV, which was close to the nominal Nernst potential of K^+^ in the experimental conditions. The test pulses ranged from −140 to +40 mV. Before the test pulses, the membrane potential was set to −120 mV. **(D)** Average I_TAIL_–V_ACT_ for the mutant R198H coexpressed with K_V_7.3. **(E)** K^+^ current recording from oocytes coexpressing the R198Q mutant along with K_V_7.3 using the same protocol in C. **(F)** Average I_TAIL_–V_ACT_ plots for the mutant R198Q coexpressed with K_V_7.3. Solid lines in B, D, and F are the Fermi–Dirac function fitted to each average I_TAIL_–V_ACT_ plot. **(G)** Average weighted V_1/2_ for each of the WT and the mutants R198H and R198Q each coexpressed with the K_V_7.3. The weighted V_1/2_ was calculated from the parameters yielded from fitting a double Fermi–Dirac function to the I_TAIL_–V_ACT_ from individual experiments. The solid lines correspond to a linear regression of the V_1/2_ as a function of pH_EXT_. We used a linear function to merely characterize the changes in V_1/2_ not assuming any type of mechanism. **(H)** Average total apparent charge associated with gating. Statistical test performed using the WT as reference. *T* test confidence intervals are color coded. For WT: *n* = 5–9; for R198H: *n* = 8–12; for R198Q: *n* = 6–14.

In the presence of K_V_7.3, the voltage dependence of K^+^ currents activation was less resilient to changes in pH_EXT_ than the K_V_7.2 channel expressed alone (Fig. 7, A and B). For the WT pair, the voltage dependence changed by a rate of −5 ± 1 mV/pH unit (*n* = 5–7; Fig. 7 G), becoming slightly more positive as the pH_EXT_ becomes acidic (Fig. 7, left). In contrast, the voltage dependence of activation of the mutants R198H (Fig. 7, C and D) and R198Q (Fig. 7, E and F) retained strong pH sensitivity. The mutant R198H shifted its voltage dependence −17 ± 2 mV/pH unit (*n* = 8–10; Fig. 7 G) like the mutant R198H expressed alone. Similarly, the mutant R198Q displayed a shift rate of −14 ± 1 mV/pH unit (*n* = 6–12; Fig. 7 G). For the three cases, changing pH_EXT_ barely altered the total apparent charge associated with activation (Fig. 7 H). These observations suggested that the modulation of the activation voltage dependence might be an intrinsic property of each subunit as the gained-by-mutation pH_EXT_ sensitivity remained in their activity when coexpressing the K_V_7.3 subunit.

Regarding deactivation, the coexpression of K_V_7.3 did not prevent the pattern observed above. K^+^ currents from oocytes expressing K_V_7.2-WT and K_V_7.3 showed minor changes in their deactivation kinetics as a function of pH_EXT_. In a remarkable contrast, the deactivation kinetics of each of the mutants K_V_7.2-R198H and K_V_7.2-R198Q coexpressed with K_V_7.3 were sensitive to pH_EXT_. In both cases, increasing pH_EXT_ strongly decreased the rate of deactivation (Fig. 7, A, C, and E). These observations indicated that the coexpression of the K_V_7.3 subunit could not override the effect of neutralizing the charge at position 198.

### The countercharge E130 is not responsible for the pH_EXT_ sensitivity

**Figure 8.**
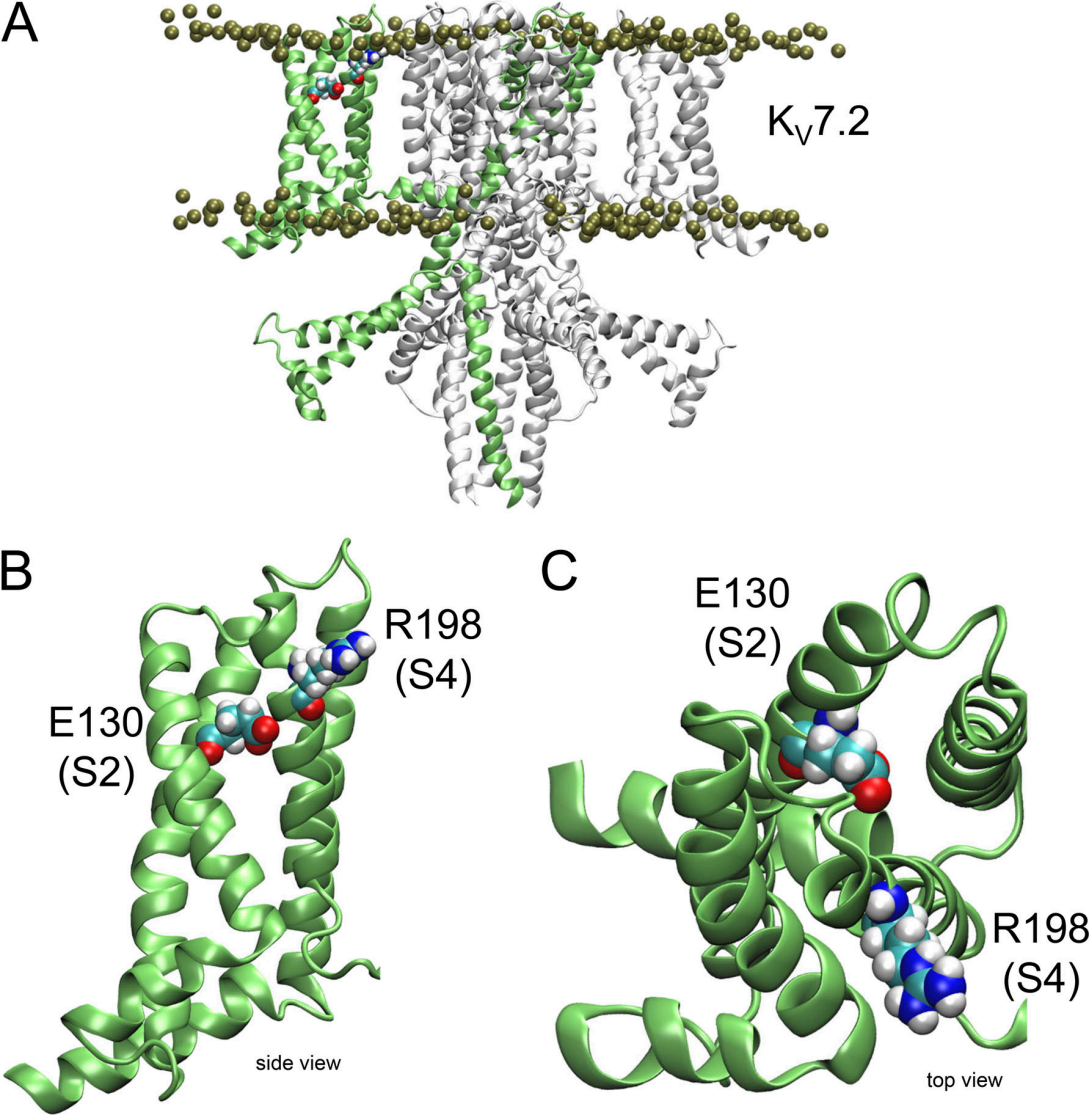
**Structural model of K**_**V**_**7.2 derived from the cryo-EM structure 7CR3. (A)** Full view of the tetrameric model embedded in a POPC bilayer (phosphate group depicted as tan spheres). One of the subunits is highlighted in green. **(B and C)** Detailed side (B) and top (C) views of VSD’s backbone from the highlighted subunit in A. Residues E130 and R198 are depicted as van der Waals spheres.

**Figure 9.**
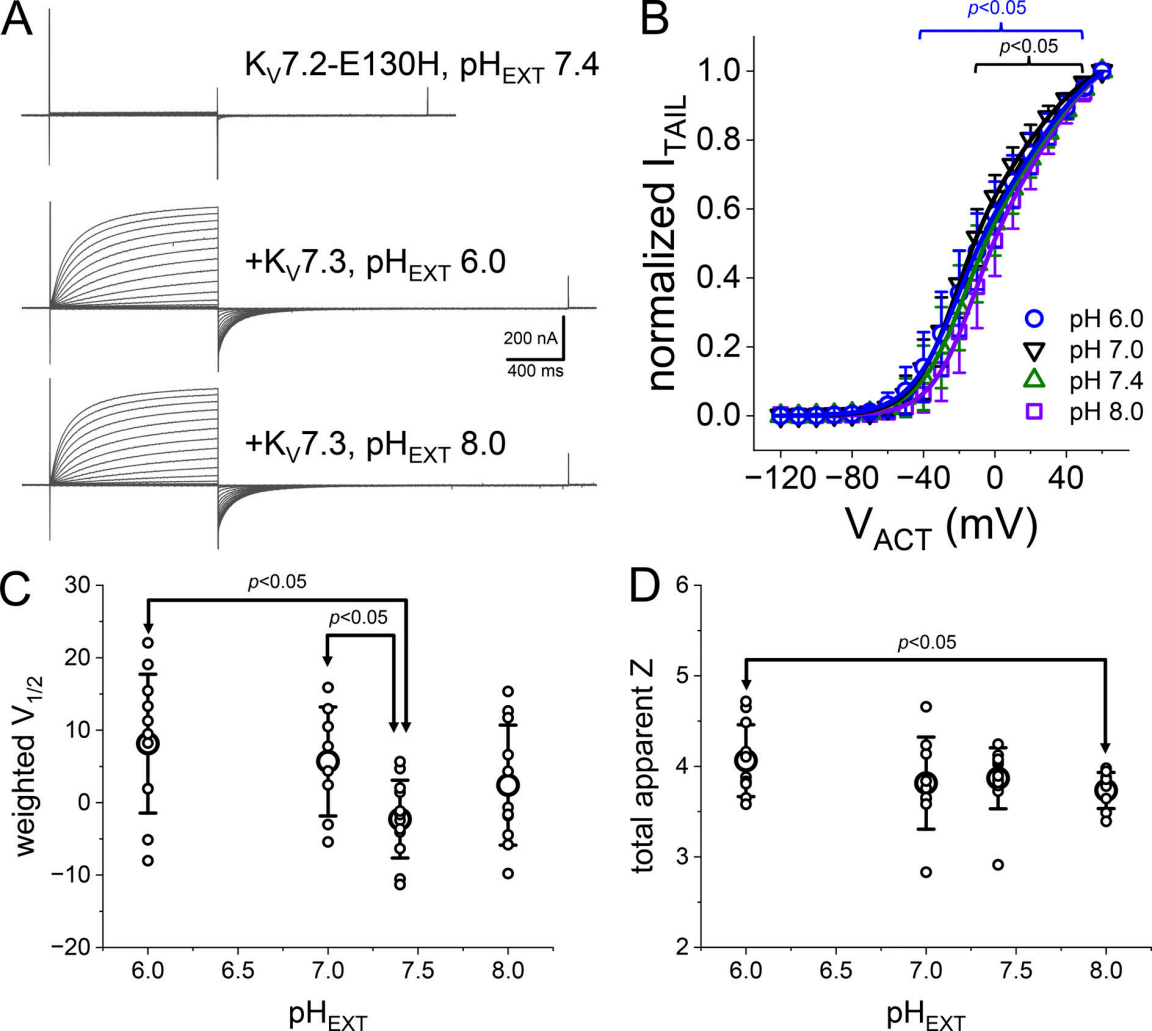
**K**^**+**^ **currents from oocytes expressing the mutant E130H. (A)** Examples of currents recorded from oocytes expressing the mutant E130H alone (top) and with K_V_7.3 (middle and bottom). **(B)** Average I_TAIL_–V_ACT_ plots for the mutant E130H coexpressed with K_V_7.3 (*n* = 9–12). Currents recorded at pH_EXT_ 6.0, 7.0, 7.4, and 8.0. **(C and D)** Average weighted V_1/2_ and average total apparent charge calculated from individual normalized I_TAIL_–V_ACT_ plots.

Observing that mutant R198Q was highly sensitivity to changes in pH_EXT_ suggested that the alterations of the voltage dependence for activation and deactivation kinetics did not emerge primarily from the altering of the protonation/electrostatic status of the residue at position 198. This led us to propose that other residues are responsible for the pH_EXT_ sensitivity acquired by the mutant channels. Among other potential candidates, we focused on one of the negative charges commonly found in the S2 segment of the VSD of channels, as suggested by reviewers of this work. To explore this idea, we looked at one of the recent cryo-EM-derived structural models reported for K_V_7.2 (Li et al., 2021). This model was incorporated in a membrane using Charmm-Gui (https://www.charmm-gui.org/), equilibrated and relaxed in a 10-ns simulation using NAMD (University of Illinois at Urbana-Champaign). We notice that glutamate at position 130 (E130) was close to arginine 198 of the model (Fig. 8). This suggested that titration of this acidic residue could be responsible for the acquired pH_EXT_-sensitivity of the mutants R198H and R198Q. We reasoned that the positive charge carried by R198 interacts with the negative charge of E130 in the down/deactivated state of the VSD. If the charge in position 198 is not present, then the putative interaction between E130 and R198 will not exist, favoring the activated state of the VSD. If this is the case, then removing the negative charge borne by residue 130 should have a similar effect than removing the charge at residue 198. To assess this hypothesis, we used the same approach described above and recorded K^+^ currents from oocytes expressing a mutated K_V_7.2 in which the residue E130 was replaced with a histidine. The idea was that if the charge of residue 130 is important, then titration by changing pH_EXT_ will allow modulation of the activity of the channel by protonation. Yet, we observed that the mutant E130H was not readily expressed in our oocytes (Fig. 9 A, top). This strongly suggested that the mutation E130H was not tolerated. This was surprising because channels bearing mutations like E130R and E130C can produce currents (Soldovieri et al., 2019). Nonetheless, we did not explore this issue any further. Instead, we proceeded to coexpress the mutant E130H with K_V_7.3. It has been well-established that the expression of K_V_7.3 alone in *Xenopus* oocytes results in small K^+^ currents (Wang et al., 1998; Etxeberria et al., 2004). Thus, if the co-expression with the mutant E130H and K_V_7.3 produced currents, that would mean that the presence of K_V_7.3 boosted the expression of the mutant subunit. Luckly, we observed a robust expression of K^+^ currents when expressing these constructs together, suggesting that the presence of the sister subunit compensated for the structural deficiency introduced by the mutation. Beyond this, we observed that the mutation did not increase the pH_EXT_ sensitivity of the channel (Fig. 9 A, middle and bottom). In fact, the average normalized I_TAIL_–V_ACT_ plots at different pH_EXT_ overlapped (Fig. 9 B). Furthermore, fitting a double Fermi–Dirac distribution to individual I_TAIL_–V_ACT_ shows that the average weighted V_1/2_ only changed at a rate of −4 mV per unit of pH_EXT_ (*n* = 9–12; Fig. 9 C). This was like what we observed with the pair K_V_7.2/K_V_7.3. Finally, changes in pH_EXT_ barely altered the apparent total charge associated with the voltage dependence of activation (Fig. 9 D). These combined observations indicated that E130 was not involved in changing the voltage sensitivity of the channel as a function of pH_EXT_ and that it only shifted the overall voltage dependence for activation by about +20 mV.

**Figure 10.**
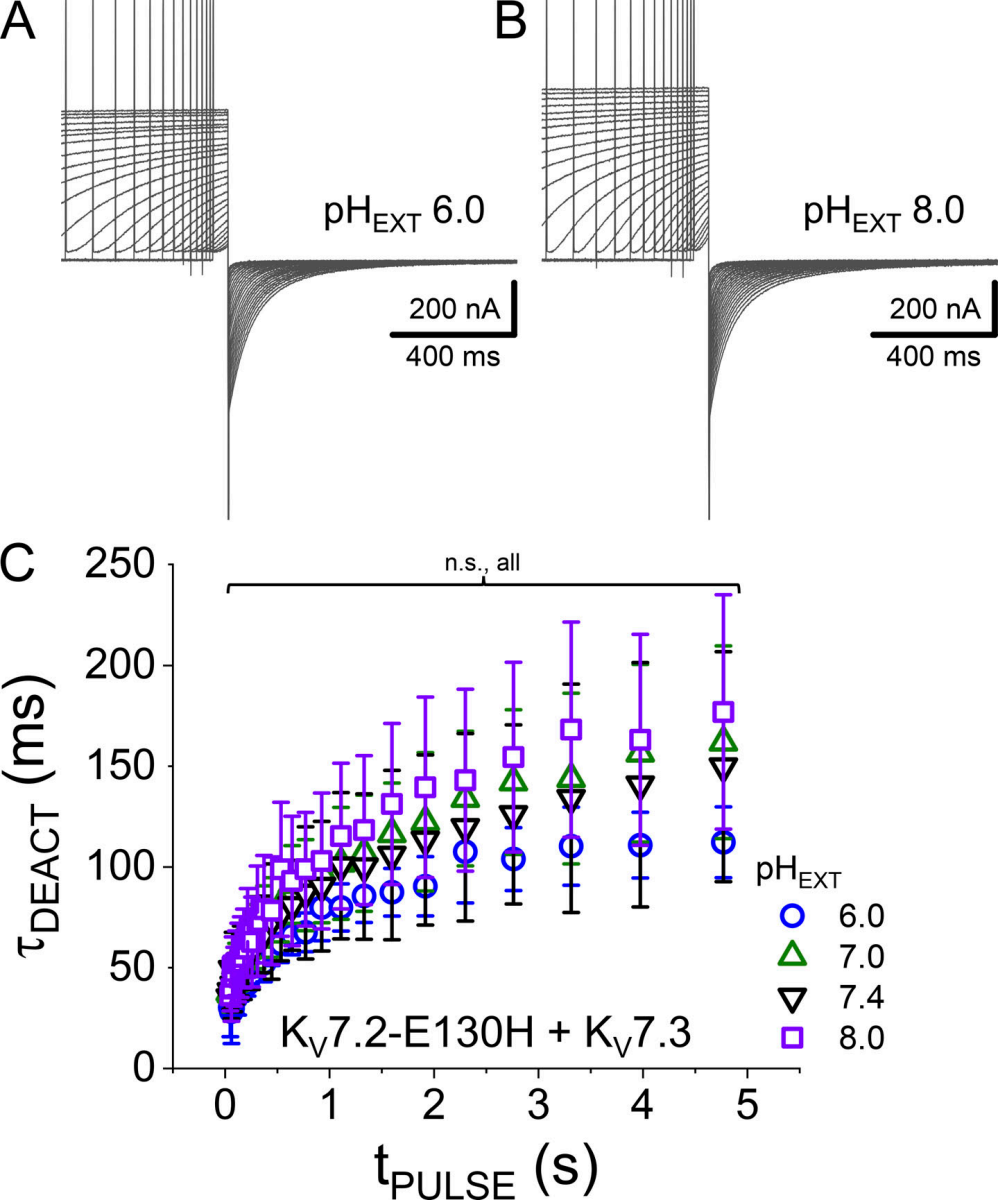
**Deactivation of the mutant E130H coexpressed with K**_**V**_**7.3. (A and B)** Examples of recordings of K^+^ current deactivation at pH_EXT_ 6.0 (A) and 8.0 (B) following activation pulse of variable duration to +40 mV. Deactivation was driven by a pulse to −105 mV. **(C)** A double exponential function was fitted to the deactivating currents (not shown). The yielded parameters were used to generate average τ_DEACT_–t_PULSE_ plots at different pH_EXT_. (pH_EXT_ 6.0: *n* = 5; 7.0: *n* = 3; 7.4: *n* = 6; 8.0: *n* = 5).

In addition to the lack of effect of change in pH_EXT_ on the activation of the mutant E130H, we also observed the deactivation was also not pH_EXT_ sensitive (Fig. 10 A). Fitting a double exponential function to the deactivating currents shows that changes in pH_EXT_ did not alter the τ_DEACT_–t_PULSE_ relationships (Fig. 10 C).

## Discussion

Here, we have shown that two charge-neutralizing mutations of arginine 198 asymmetrically affect the activation and deactivation of the human K_V_7.2 channel. On one hand, removal of the charge at position 198 by mutation caused a shift in voltage dependence for activation, which is consistent with a previous report (Millichap et al., 2017). This shift also caused a concomitant yet small alteration of the activation kinetics. On the other hand, the charge-neutralizing mutations produced remarkable changes in the deactivation kinetics and conferred a higher pH_EXT_ sensitivity to the mutant channel’s activity. This asymmetrical effect on pH_EXT_ sensitivity highlights the notion that, at least for K_V_7.2 and K_V_7.2/K_V_7.3 channels, the activation and deactivation processes follow distinct pathways (Corbin-Leftwich et al., 2016; Villalba-Galea, 2020).

The residue R198 is in the fourth (S4) segment of the VSD, where it is the outermost arginine of the segment (Li et al., 2021). In that position, R198 could function as one of the sensing charges of the channel’s VSD. Adding and removing charges in the S4 segment can cause changes in voltage dependence, kinetics, and the appearance of additional conductances (i.e., “omega”/“gating pore” currents), among other loss-of-function and gain-of-function alterations (Ahern and Horn, 2004; Starace and Bezanilla, 2004; Sokolov et al., 2007; Catterall, 2010; Zhao and Blunck, 2016; Gamal El-Din et al., 2021). Varying the residue composition of the voltage sensor of a channel, particularly S4 residues, has resulted in changes in the activity of channels that cannot be explained only in terms of basic electrostatic (Starace et al., 1997; Starace and Bezanilla, 2001; Ahern and Horn, 2004; Sokolov et al., 2007; Yang et al., 2007, 2011; Gagnon and Bezanilla, 2009; González-Pérez et al., 2010). These types of alterations in the properties of the channels are due to remodeling of the sensor so that changes in local hydrophobicity, hydration, and steric hindrance play a critical role along with changes in the electrostatic of each mutated residue. Here, the introduction of a histidine at the top of the S4 segment (mutation R198H) granted the channel with high pH_EXT_ sensitivity. Decreasing pH_EXT_ shifted the voltage dependence of activation of the charge-neutralized mutants to a more positive potential, while increasing pH_EXT_ had the opposite effect. To our surprise, it was not the ability to titrate the histidine with protons but the lack of the charge itself that conferred high pH_EXT_ sensitivity as the non-titratable mutation R198Q displayed a pH-dependent behavior like the mutant R198H.

A recent study showed that the spasm/epilepsy-related mutation R198Q causes a shift in voltage dependence to more negative potential, while decreasing the slope of the current amplitude-vs.-membrane potential curve, supporting the idea that R198Q is likely the first sensing charge. However, careful fitting of the current-vs.-voltage relationship (i.e., I_TAIL_–V_ACT_ plots in Fig. 2) indicated that the “steepness” of these curves did not change with pH_EXT_, strongly suggesting that R198 does not play a role as a sensing charge. Although the “slope” is a poor indicator of the number of charges involved in conferring voltage dependence to channels (Bezanilla and Villalba-Galea, 2013), the fact that changing the pH_EXT_ did not alter the steepness of the I_TAIL_–V_ACT_ plots supports the idea that R198 might not be playing a role as a first sensing charge.

Another argument against, yet not proving, the idea that R198 is the first sensing charge in the VSD of K_V_7.2 is the absence of inwardly rectifying currents at negative potentials. If R198 was the first sensing charge, the mutation R198H could open a conduction pathway, producing a down-state current through the VSD like in the case of sodium-selective voltage-gated (Na_V_) channels (Sokolov et al., 2007), the K_V_ channel *Shaker* (Starace et al., 1997; Starace and Bezanilla, 2001), the voltage-sensitive phosphatase (Ci-VSP; Villalba-Galea et al., 2013), and K_V_7.3 channels (Gamal El-Din et al., 2021). Again, the absence of such currents does not prove that R198 is not the first sensing charge of the VSD’s S4 segment. Nonetheless, we propose that R198 plays a critical role in the modulation of channel activity while not directly participating in voltage sensing.

### Asymmetric effect of charge-neutralizing mutations of R198 on activation and deactivation

A remarkable feature of both R198H and R198Q is that the effect of pH_EXT_ on their deactivation kinetics seems to be “out of proportion” with respect to that on the activation kinetics. In the case of the mutant R198H, changes in pH_EXT_ modestly affected the activation kinetics compared with the changes observed in deactivation. For activation, the effect of changes in the pH_EXT_ seems to cause electrostatic effects, at least for the mutant R198H. In fact, replotting the values of τ_FAST_, τ_SLOW_, and *f*_1_ for this mutant (Fig. 3, G–I) and adding a bias of −17 mV/pH_EXT_ units made those relationships almost overlap (Fig. S1). This suggested that the protonation of R198H mostly produced an electrostatic effect on voltage sensing. However, the “protonation hypothesis” does not explain what happened with the mutant R198Q. Thus, we can only conclude at this point that the presence of a positively charged residue in position 198 allows the channels to overcome the stabilization of the open channel when increasing pH_EXT_.

Regarding deactivation, the kinetics of this process for the charge-neutralized mutants displayed larger differences of four and sevenfold as a function of pH_EXT_. This indicated that the process of activation and deactivation follow distinct pathways so that they are not each other’s reverse processes. This is consistent with previous observations, in which the effects of K_V_7 channel modulator retigabine and the regulation by the phosphoinositide PI(4,5)P_2_ seem to differentially affect activation and deactivation (Villalba-Galea, 2020).

It is not clear what the mechanism underlying the asymmetrical effect of pH_EXT_ on the channel’s kinetics is. Here, we propose that at high pH_EXT_ the open conformations of the channel become resilient to closing. However, the presence of a charge at position 198 allows for an effective voltage-driven deactivation of the channel. Alternatively, a charged residue in position 198 could be part of a network of salt bridges, making the channel less susceptible to protonation. The absence of this charge disrupts this hypothetical network, causing the rearrangement of the VSD and so changing voltage dependence for activation. Although distinguishing between these and other mechanisms escapes the scope of this study, we proceeded to alter one obvious candidate residue that could be a member of this putative network. That residue E130 is one of the negative charges found in S2 of the VSD, it could be interacting with R198 in the deactivated conformation of the VSD since E130 faces the extracellular crevice of the domain (Fig. 8). We observed that mutant E130H was not readily expressed in oocytes. This suggested, as mentioned before, that the protein was not properly folded or simply was not functional. Identifying the reason for the lack of current also escapes the scope of this work. However, we observed that coexpression with K_V_7.3 rendered robust K^+^ currents in oocytes. This strongly suggested that the presence of the sister subunit was able to “mitigate” the effect of structural and functional deficiencies caused by the mutation. Furthermore, we observed that the resulting channels displayed a voltage dependence for activation that was shifted to more positive potentials with respect to the WT pair. Finally, we observed that the deactivation kinetics was unaltered by changes in pH_EXT_. This was surprising to us, given the position of the residue in the VSD. We speculate that it is possible that the introduced histidine was always protonated in the range of pH_EXT_ used in this study. However, we observed with the R198 mutant that the charge of the residue does not seem to be a determinant factor in the gained modulatory effect by pH_EXT_. This suggests that conformational changes leading to the stabilization of the open conformation of the channel are highly pH_EXT_ dependent and that the charge in position 198 disrupts that stabilized open conformation of the channel.

### A positively charged residue in position 198 disrupt pH_EXT_-dependent open channel stabilization

**Figure 11.**
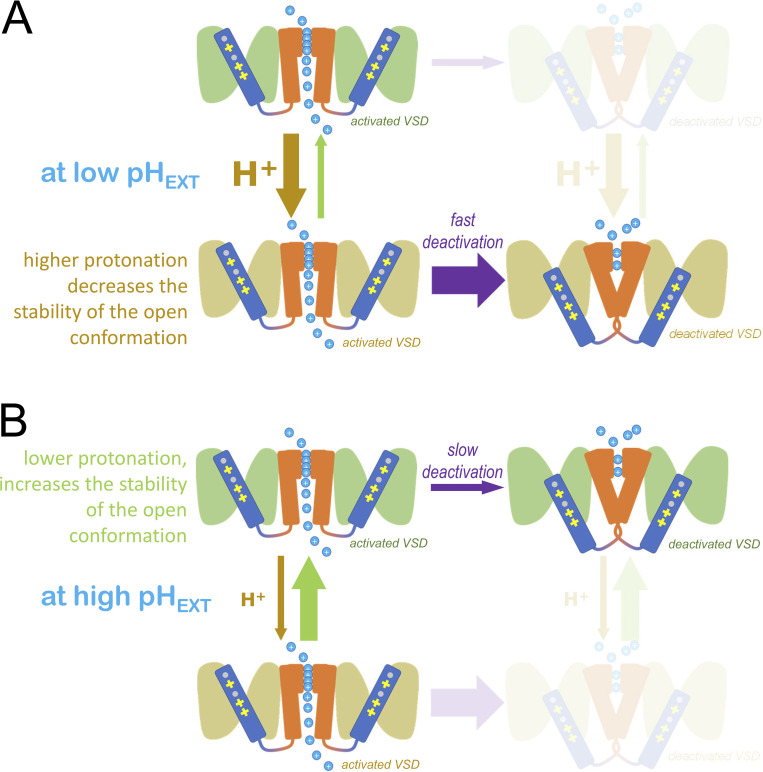
**Kinetic scheme providing an interpretation of the findings on the deactivation kinetics of the mutant R198Q.** Following activation, K_V_7.2 channels tend to become resilient to close as they remain activated. This shows a reduction in the rate of deactivation, making it slower. The activated mutant R198Q becomes more stable as the channels remain activated by depolarization (Fig. 6 D). This happened at any pH_EXT_. However, at low pH_EXT_ deactivation was faster for any duration of the activating pulse than those observed at higher pH_EXT_ (e.g., Fig. 6 D, pH_EXT_ 6.0, blue circles, and pH_EXT_ 8.0, purple squares, respectively). **(A and B)** This strongly suggests that the conformation of the highly protonated open channels (A) is less stable than that of the channels that are less protonated (B). Thus, at low pH_EXT_, the channels readily deactivate, while at higher pH_EXT_, the channels deactivate slowly.

At first, we thought that the lack of a positive charge in position 198 was facilitating the upward movement of the S4 segment at negative potentials simply by decreasing the electrical force driving the segment toward the intracellular side (“down”). In fact, titrating the introduced histidine in the mutant R198H made deactivation faster at low pH_EXT_ than in a more alkaline environment. However, the mutant R198Q showed similar behavior, indicating that changes in the deactivation rate were associated with alteration in the stability of the open channels. In general, what we proposed is that the open-channel conformation becomes destabilized at acidic pH_EXT_. Accordingly, the rate of deactivation increases at acidic pH_EXT_ (Fig. 11 A). Conversely, the open conformation of the channels is further stabilized at alkaline pH_EXT_, leading to a decrease in the rate of deactivation (Fig. 11 B).

What is the role of R198 in open channel stabilization? According to the work presented here, a positive charge in position 198 can disrupt the stabilization of the channel’s open conformation. Increasing pH_EXT_ caused the channels to become resilient to close as the deactivation became slower than that at lower pH_EXT_. This is illustrated in the case of the mutant R198Q. Thus, the electrical properties of residue 198 are not sufficient to entirely explain the effect of pH_EXT_ on the charge-neutralized mutants studied here.

### A final thought: Potential effect of the mutation R198Q on neuronal hyperexcitability

The pH_EXT_-sensitive behavior of the mutant R198Q opens a new door to the understanding of its role in disease. Our initial question driving this project was: “How can a mutation that increases the activity of K_V_7.2 channels produce hyperexcitability?” This apparent paradox was intriguing. We argue that the changes in both voltage dependence and deactivation kinetics of the mutant R198Q could contribute to the prolongation of seizures/spasms. During high electrical activity, acidification of the extracellular environment in neuronal tissue can occur due to high electrical activity (Raimondo et al., 2015; Sulis Sato et al., 2017). In this situation, the low pH_EXT_ will decrease the stability of the open mutant channel as well as shift its activation voltage dependence toward positive potential. These two actions would effectively reduce the basal K^+^ conductance in the plasma membrane. Such a decrease could facilitate the triggering of action potentials. This would lead to further local acidification of the extracellular environment and eventually unchaining out-of-control activity, a seizure. In these terms, we propose that the mutation can be epileptogenic by enhancing excitability following local extracellular acidification. This hypothesis challenges the old notion that systemic acidosis ceases seizures, while alkalosis triggers them (Xiong et al., 2008; Cheng et al., 2021). In this case, we argue that the mutant channel is not responsible for the ictal stage of a seizure, instead the acidification drives a decrease in its activity and would further prolong high electrical activity.

**Figure 12.**
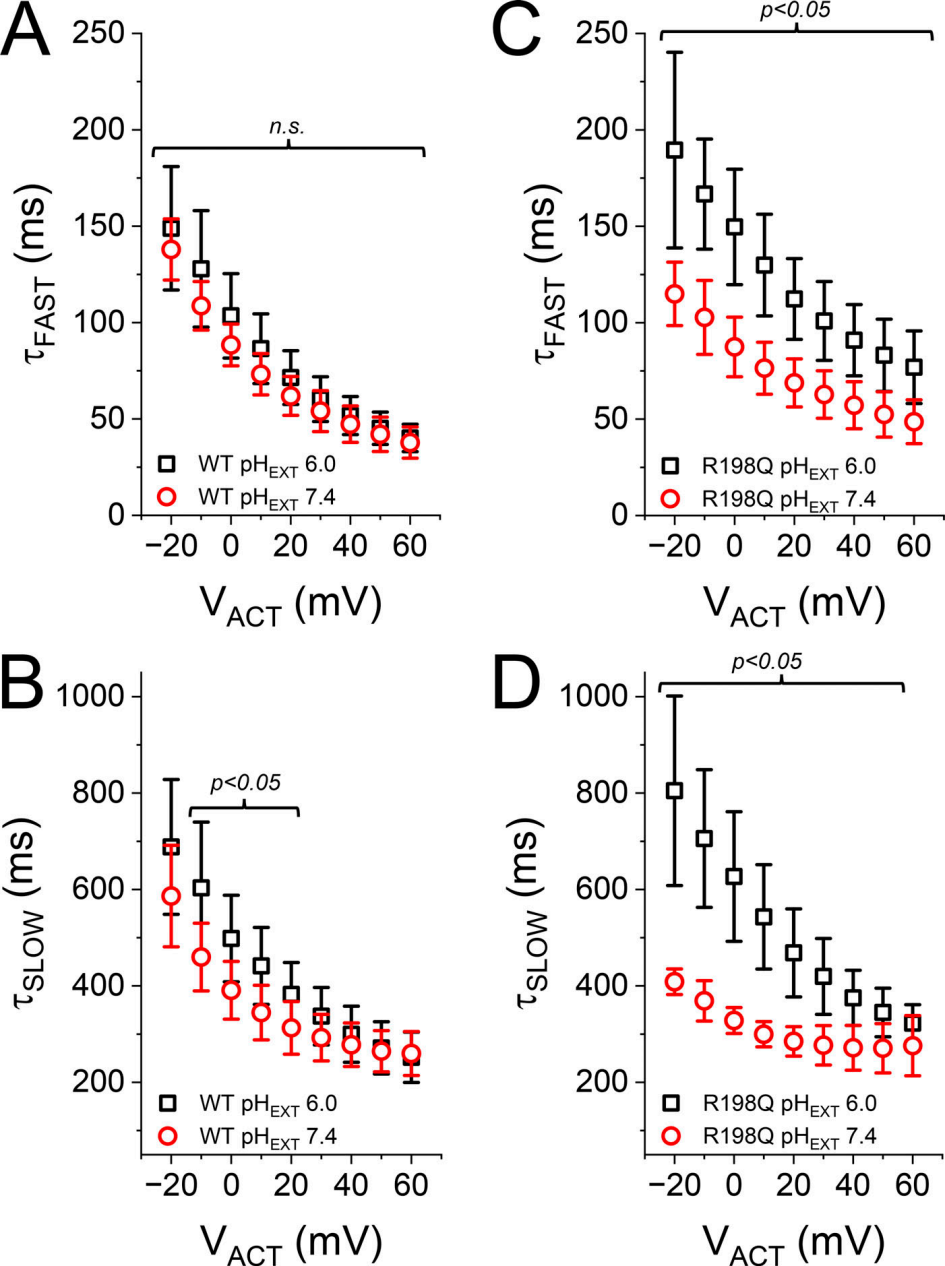
**Activation kinetic of the heteromeric K**_**V**_**7.2/K**_**V**_**7.3 (WT) and K**_**V**_**7.2-R198Q/K**_**V**_**7.3 (R198Q) channels. (A–D)** The *n*-powered two-exponential function was fitted to the activation phase of K^+^ currents recording from oocytes expressing the WT K_V_7.2 (A and B) and the mutant R198Q (C and D) coexpressed with K_V_7.3. In both cases, the holding potential was set to −90 mV. The average fast (A and C) and slow (B and D) time constants were calculated from the parameters yielded by the fitting on individual experiments (WT: *n* = 9; R198Q: *n* = 8).

Again, how can the mutation K_V_7.2-R198Q lead to hyperactivity? Arguably, the number of open K_V_7 channels open would not change through the course of a single neuronal action potential (nAP). This is because the channel’s kinetics for activation and deactivation is slower than the time course of a nAP. In contrast, during a burst of nAP, the fraction of open K_V_7 channels should increase. Consistent with this idea, K_V_7 channels have been shown to contribute to the generation of after hyperpolarization potentials (Tzingounis and Nicoll, 2008; Kim et al., 2012; Larsson, 2013) among other channels (Cloues and Sather, 2003). In the case of the WT pair K_V_7.2/K_V_7.3, successive depolarizations during a burst of nAP would increase the number of K_V_7.2/K_V_7.3 channels that open, making the K^+^ conductance more robust as nAP continue to fire. This effect on K^+^ conductance will drive the termination of the burst. In contrast, for the mutant pair K_V_7.2-R198Q/K_V_7.3, the firing of successive nAP would lead to a decrease in pH_EXT_ (Raimondo et al., 2015), causing an increase in the rate of deactivation, thereby making the K^+^ conductance less robust. In addition to the increase of the deactivation rate, the mutant K_V_7.2-R198Q/K_V_7.3 activation becomes slower (Fig. 12, C and D) as the pH_EXT_ decreases—this is not the case for the WT pair (Fig. 12, A and B). This suggests that the contribution of the mutant K_V_7.2-R198Q/K_V_7.3 channel to the K^+^ conductance would decrease as pH_EXT_ drops due to AP firing.

Another factor to consider is the contribution of Na_V_ channels. Having a hyperpolarized membrane potential would recover Na_V_ channels from resting state inactivation, making them available for activation, so increasing excitability. Using a simple model for AP in oocytes (Corbin-Leftwich et al., 2016, 2018), our preliminary data show that in the presence of the R198Q mutant, the maximum rate of depolarization during an AP is higher than those observed in the presence of the WT K_V_7.2 channels. Furthermore, our preliminary data also show that the resting potential hyperpolarizes in the presence of the mutant channels and that the threshold potential (V_TH_) for triggering APs becomes more negative. This is different than what we reported on the effect of retigabine using a similar AP model in oocytes. In that case, retigabine hyperpolarized the membrane but left the V_TH_ unaltered (Corbin-Leftwich et al., 2016). Thus, changing the V_TH_ may be a key factor in the underlying mechanism for hyperexcitability caused by mutations like R198Q. Therefore, we hypothesize that the effect of the mutation R198Q goes beyond changes in the resting membrane potential and repolarization, having a direct impact on the activity of other channels (i.e., Na_V_ channels), as the membrane potential couples their activity.

## Supplementary Material

Review History

## Data Availability

All data are available in the main text or the supplementary materials.
